# Validity of the Walter Reed Visual Assessment Scale to measure subjective perception of spine deformity in patients with idiopathic scoliosis

**DOI:** 10.1186/1748-7161-1-18

**Published:** 2006-11-08

**Authors:** Sonia Pineda, Juan Bago, Carmen Gilperez, Jose M Climent

**Affiliations:** 1 Department of Physical Medicine and Rehabilitation, Hospital Vall d'Hebron, Barcelona, Spain; 2 Spine Unit, Hospital Vall d'Hebron, Barcelona, Spain; 3 Department of Physical Medicine and Rehabilitation, Hospital Universitario, Alicante, Spain

## Abstract

**Background:**

The Walter Reed Visual Assessment Scale (WRVAS) was designed to allow idiopathic scoliosis patients to describe their perception of their deformity. In a previous stduy, the scale has shown good correlation with magnitude of the curve

**Methods:**

The study included 70 patients (60 women and 10 men), mean age 19.4 years (range 12–40), with idiopathic scoliosis. Each patient filled out the WRVAS and the SRS-22 questionnaire. Thoracic and lumbar curve angles were determined in standing X-rays and the largest was named Cobbmax. WRVAS internal consistency was assessed with Cronbach's alpha. Correlation coefficients were calculated between Cobbmax and the various WRVAS questions, and Cobbmax and the SRS-22 scales. The correlation between the WRVAS and SRS-22 was also determined

**Results:**

Mean magnitudes were thoracic curve, 36.6° and lumbar curve, 33.2°; average Cobbmax was 37.9°. The mean total WRVAS score was 15.6. Mean scores for the various SRS-22 scales were function 4.6, pain 4.3, self-image 3.7, mental health 4.2, and total score 84.1. Internal consistency for the WRVAS was excellent (Cronbach's alpha, 0.9), and there were no signs of collinearity among the seven questions (tolerance range 0.2–0.5). All the items on the WRVAS correlated significantly with Cobbmax (correlation coefficients, 0.4 to 0.7). The correlation between the total WRVAS and total SRS-22 score was -0.54 (P = .0001) and between WRVAS total score and SRS-22 image domain score was -0.57 (p = 0.0001)

**Conclusion:**

The WRVAS showed excellent internal consistency and absence of collinearity. There was a highly significant correlation between the results of the test and the magnitude of the deformity. The WRVAS correlated significantly with the SRS-22 image scale. The WRVAS is a valid instrument to assess scoliosis patients perception of their deformity

## Background

One of the main features of scoliosis is the cosmetic defect caused by the three-dimensional deformity. This problem is a major concern both for patients and physicians [1,2]. To facilitate the management of this aspect of the disease, several methods have been devised to measure the magnitude of the deformity. These include surface contour mapping techniques such as Moiré topography, based on the projection and observation of shadows on the back [3,4] and procedures that use optoelectronic methods, such as the ISIS [5] or Quantec surface imaging systems [6,7]. Nevertheless, all these methods have serious drawbacks: they require expensive equipment that is complex to manage and their reliability is debatable, since they depend on the patient's posture and the expertise of the operator. Moreover, the measures obtained are difficult to interpret for physicians who are not specialists in the field. Other authors have attempted semiquantification of the magnitude of the deformity by observers who score various visible aspects of the deformity [8,9]. This method is only useful for research purposes, since in clinical practice it would require that the patient be assessed by several examiners. Another approach is to request the patient's personal impression of the deformity. Some instruments that measure quality of life, such as the CAVIDRA [10] profile or the SRS-22 [11-14] questionnaire contain scales to determine self-perception of the body image. However, the correlation between self-image scales and the radiological magnitude of the curve is weak, indicating poor agreement between the patient's perceived image and the magnitude of the deformity. Consequently, there is no clear evidence that the perceived body-image scales actually correlate with the deformity, itself.

The Walter Reed Visual Assessment Scale is a new option among these efforts. It consists of a visual test including seven items that deal with various aspects of the deformity. Each question has a set of five figures that represent degrees of severity of the deformity: spinal deformity, rib prominence, lumbar prominence, thoracic deformity, trunk imbalance, shoulder asymmetry and scapular asymmetry. The test can be completed by the patient or by an external evaluator. In the single report concerning this questionnaire [8], the data provided showed a good correlation between the responses given by patients and those of their parents, as well as a good correlation between the test scores and the magnitude of the scoliotic curve.

The questionnaire is simple; it can be filled out and scored rapidly. This fact suggests that it might be useful for daily clinical practice. Nevertheless, the study mentioned above does not provide data on the metric properties of the test. Because of the high potential interest of the scale, we believe this information should be available before issues such as its practical utility are investigated.

The main objective of this study is to analyze the internal consistency and the construct validity of the Walter Reed Visual Assessment Scale for scoliosis.

## Methods

A cross-sectional study was conducted to analyze the homogeneity and construct validity of the questionnaire. Patients were consecutively recruited in two different centers. The inclusion criteria were a diagnosis of idiopathic scoliosis, age between 10 and 40 years and acceptance to participate in the study. A total of 70 patients (60 women and 10 men) with a mean age of 19.4 years (range, 12 to 40 years) were included. There were 40 patients under or equal to 18 years and 30 patients older. All had idiopathic scoliosis: 42 were being actively treated with bracing, 8 were growing adolescents under observation, 15 were at the end of the growth period and had completed treatment, and 5 patients were awaiting surgery. Each patient filled out the SRS-22 questionnaire and the Walter Reed Visual Assessment Scale (WRVAS). The magnitude (Cobb angle) of the thoracic curve and thoracolumbar/lumbar curve on radiographic study were recorded. The mean magnitudes of the thoracic curve and lumbar curve were 36.6° ± 19.5 and 33.2° ± 12.2, respectively. To simplify the statistical calculation, the radiological magnitude was summarized in a variable representing the greatest Cobb angle of the two curves recorded, termed the *Cobbmax*. The average Cobbmax was 37.9° ± 18.4.

### SRS-22 questionnaire

This instrument is specifically dedicated to the assessment of quality of life in patients with idiopathic scoliosis. Its current form was designed by Asher et al. [11-14] and a completely validated adapted version in Spanish is now available [15,16]. It consists of 22 questions that represent four scales containing five questions each: pain, function-activity, self-image, and mental health. The remaining two questions refer to satisfaction with the treatment received and were not used in the present study. Each question is scored from 1 (worst situation possible) to 5 (best situation possible) and the results are presented as the mean of each scale (sum of the questions/n° of questions answered). The sum of all the questions ranges from 20 to 100.

### Walter Reed Visual Assessment Scale [8]

**Figure 1:**
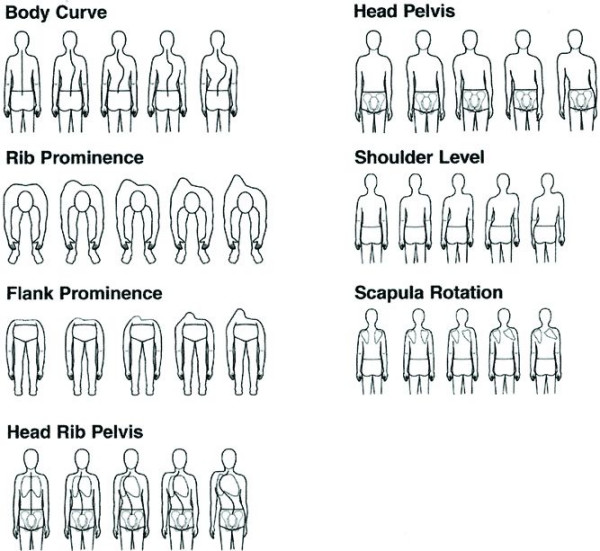
**The Walter Reed Visual Assessment Scale**. (used with permission from Sanders et al.^18^).

This instrument includes a group of figures (Fig. 1) representing seven aspects of the deformity: item 1, spinal deformity; item 2, rib prominence; item 3, lumbar prominence; item 4, thoracic deformity; item 5, trunk imbalance; item 6, shoulder asymmetry; and item 7, scapular asymmetry. Each aspect is shown with five levels of increasing severity of the deformity that are scored from minimum (1) to maximum (5). Results are presented as the sum of the seven questions. The figures present an image of the individual as seen from behind. Hence, the WRVAS measures the subjective perception of the deformity from this perspective, or, in other words, how the patient feels that others see his or her back.

### Statistical analysis

The mean and standard deviation (SD) were determined for each of the WRVAS questions; the distribution of the scores served to establish the floor and ceiling effects (percentage of cases with minimum and maximum scores).

Internal consistency of the WRVAS was determined with Cronbach's alpha coefficient. This tool assesses the homogeneity of the items on a questionnaire; that is, whether the items included measure the same latent variable and score it in the same direction. Optimum Chronbach alpha scores are between 0.7 and 0.9.

When the various items on a scale present a very high correlation, there is a possibility that collinearity may be involved. Collinearity indicates that different items are very similar, i.e. that they are redundant and repetitive. The existence of collinearity was analyzed by calculating the tolerance and the variance index factor (VIF). The following were considered indicators of collinearity [17]: tolerance values less than 0.1 and VIF greater than 10 for any of the seven questions.

Construct validity was analyzed from two perspectives: discriminant validity and convergent validity. The discriminant validity of the WRVAS was determined by calculation of the simple correlation coefficient (Pearson) between the Cobbmax and the various WRVAS questions. The convergent validity was estimated with the simple correlation coefficient (Pearson) between the WRVAS questions and the SRS-22 scales.

Lastly, construct validity was also assessed by multiple regression analysis between the various items on the WRVAS and the SRS-22 self-image dimension to determine which WRVAS questions had a significant influence in the SRS-22 self-image scale.

Additionally, the correlation between the Cobbmax and the four SRS-22 scales was calculated to gain an idea of the intensity of this relationship as compared to that observed between the magnitude of the curve and the WRVAS items.

## Results

The mean and percentage of patients with a minimum (floor effect) and maximum (ceiling effect) score for each WRVAS question are shown in Table 1. The mean of the sum of the seven questions was 15.6 ± 5.

**Table 1:** Means and floor and ceiling effects of the WRVAS questions

***Question***	**Mean**	***% patients with a minimum score***	***% patients with a maximum score***
#1	2.8	0	10.1
#2	1.9	35.7	0
#3	2.0	18.6	1.4
#4	1.9	28.6	0
#5	2.5	15.7	5.7
#6	2.1	22.9	1.4
#7	2.2	25.7	1.4

*Internal consistency *of the WRVAS was excellent, with a Cronbach alpha of 0.9. No signs of collinearity were found between the seven questions (tolerance 0.2 to 0.5, VIF 1.9 to 3.8 for all the questions). Cronbach's alpha coefficient was similar in both groups of age (0.8924 in ≤ 18 years and 0.8967 in ≥ 19 years).

### Discriminant validity

The correlation coefficient between the Cobbmax and the total WRVAS was 0.69 (*P *< .0001). The coefficients of correlation with the various questions were as follows: item 1, 0.71; item 2, 0.58; item 3, 0.48; item 4, 0.56; item 5, 0.51; item 6, 0.41 and item 7, 0.56 (all, *P *< .01).

### Convergent validity

The correlation coefficients between the total WRVAS and different SRS-22 scales were: function -0.4, pain -0.41, body image -0.57 and mental health -0.43 (all p = .0001). The simple correlation coefficients between the seven WRVAS questions and the four SRS-22 scales and their statistical significance are detailed in Table 2. The correlation coefficient between WRVAS total score and SRS-22 total score was -0.54 (*P *= .0001).

**Table 2:** Correlation coefficients between WRVAS questions and SRS-22 scales

	***SRS-22 function***	***SRS-22 pain***	***SRS-22 self-image***	***SRS-22 mental health***
WR 1	-.39**	-.42**	-.55**	-.47**
WR2	-.38**	-.25	-.40**	-.32**
WR3	-.32**	-.28	-.40**	-.28
WR4	-.18	-.25	-.42**	-.31**
WR5	-.29	-.33**	-.51**	-.33**
WR6	-.30	-.34**	-.40**	-.33**
WR7	-.38**	-.36**	-.48**	-.35**

** *P *< .001

### SRS-22 patient questionnaire

Means scores (± SD) for the various SRS-22 scales were: function 4.6 ± 0.5; pain 4.3 ± 0.8; self-image 3.7 ± 0.8, mental health 4.2 ± 0.8 and total (over 100) 84.1 ± 12.4. The correlation coefficient between the Cobbmax and sum of the SRS-22 was -0.43 (*P *= .0001). The coefficients of correlation between Cobbmax and the various SRS-22 scales were: function, -0.37; pain, -0.29; self-image, -0.48 and mental health, -0.33 (all *P *< .05).

### Multivariate analysis

The dependent variable in the model was the score on the SRS-22 self-image scale and the independent variables were the WRVAS questions. The questions that entered into the equation (r = 0.63, *P *= .0001) were item 1 (spinal deformity) and item 5 (trunk imbalance), which explained 39.5% of the variance on the SRS-22 self-image scale.

## Discussion

The primary aim of the present project was to provide information on the metric properties of the WRVAS. First, the frequency distribution of the various questions comprising the instrument were analyzed to determine the floor and ceiling effects, i.e., the percentage of patients with minimum and maximum scores. The observed effects seemed adequate. The ceiling effect was notably low (0% to 10%), whereas the floor effect was somewhat higher (0% to 35%); these findings are reasonable considering that the mean magnitude of the curve was 37.9° and that in 29 patients (41% of the cases) it was less than 30°. These data are in keeping with the clinical experience, which has shown that patients are not aware of very mild curves. The percentages indicate that the test would show high sensitivity to the changes occurring with worsening of the scoliosis; an improvement following correction of the deformity would only be perceived in severe deformities, which, in fact, are those that may require surgical correction.

Second, the internal consistency of the test was assessed by determining Cronbach's alpha coefficient. The value was found to be high (Cronbach's alpha 0.9) and this raised the concern that there might be a problem of collinearity. Nevertheless, the tests to investigate collinearity excluded this possibility by ruling out that that some of the WRVAS questions might be redundant. Thus, each of the seven items can be considered to assess different aspects of the same latent variable, perception of the deformity. Internal consistency was similar in adolescents (≤ 18 years) and adults (older than 19 years).

**Figure 2:**
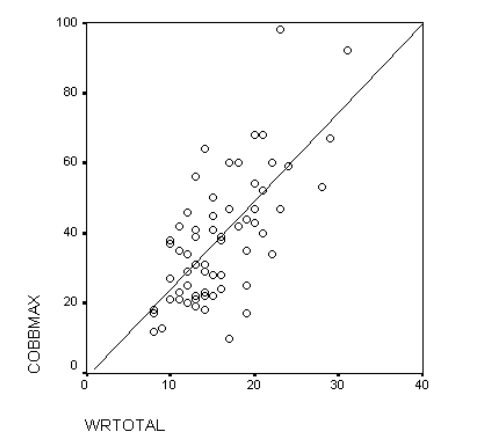
Plot of WRVAS total score and Cobbmax.

**Figure 3:**
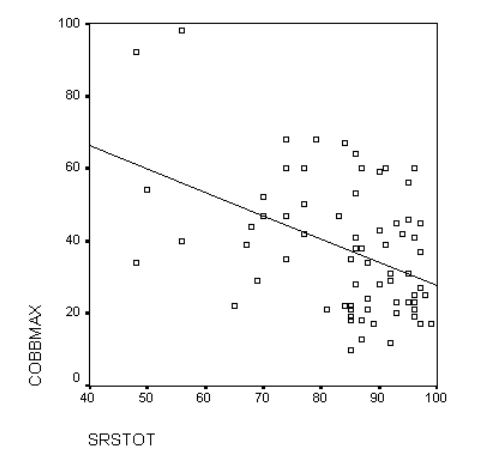
**Plot of SRS-22 total score and Cobbmax**. Comparing the slope of the regression line from figures 1 and 2, it is evident that a better correlation exists between WRVAS and Cobbmax.

The construct validity of a scale refers to the property of having appropriate relationships with variables other than those that the scale is actually measuring [18]. Construct validity is evaluated from two perspectives: discriminant validity and convergent validity. The discriminant validity indicates the capacity of the test to differentiate among the different degrees of severity of the condition. To analyze this, the WRVAS scores obtained were related with the patients' radiological magnitude of the curve. Our data showed a highly significant correlation between all the questions on the test and the Cobb angle, confirming the reported findings of Sanders et al [8]. It is noteworthy that the correlation coefficients we found between the WRVAS questions and the Cobb angle were considerably higher than those between the SRS-22 and the Cobb angle (Fig. 2 and Fig 3), a fact that indicates a higher discriminating capacity for the WRVAS.

The convergent validity assesses the relationship between the study instrument and another instrument that has been highly validated and serves as a gold standard. The convergent validity was assessed by determining the correlation between the WRVAS and the SRS-22 Patient Questionnaire. Despite its rather recent introduction [11-14], we chose the SRS-22 because it is the only instrument specifically designed to assess quality of life in idiopathic scoliosis. Moreover, it is the only instrument including a self-perception scale. It could also be of interest to compare data from the WRVAS with a General Health questionnaire such as the SF-36 questionnaire. Nevertheless, data from Lai et al. [19] suggest that self-image perception (from the SRS-22 domain) is poorly predicted by the SF-36 General Health Questionnaire. This data support the concept that appearance perception is a unique characteristic of the scoliosis and that specific instruments are better for its evaluation. The Walter Reed scale clearly evaluates the aesthetic impact of the deformity, as was demonstrated by the significant correlation between all the questions and the SRS-22 self-image scale. In contrast, correlations with pain and function were marginal (only with some questions and with coefficients <0.4). These data are in keeping with the results of Asher et al. [6], who analyzed the relationship between the SRS-22 scales and various topographic measurements obtained with the Quantec system in a group of patients treated surgically for idiopathic scoliosis. The authors found no relationship between the SRS-22 scales and the surface deformity, a fact suggesting that the relationship between the measured deformity and the perception of body image, pain and function is negligible.

Analysis of the correlations between the Walter Reed items and the mental health scale also provided notable findings. Classically, cosmetic compromise has been considered a critical factor for patients with idiopathic scoliosis. The psychological distress these patients experience is often attributed to the appearance of the trunk [9]. Nevertheless, our data do not support this impression. Only one question on the WRVAS showed a correlation coefficient greater than 0.4 with the SRS-22 mental health scale. The SRS-22 uses the same questions to assess mental health as the general health questionnaire, SF-36. With the latter instrument, Danielsson et al. [20] found no significant differences in either the metal health scale or the total mental health value in scoliosis patients undergoing bracing or surgery, when compared with an age- and sex-adjusted control group. All this evidence questions the idea that these patients regularly experience psychological conflicts, and when conflicts do manifest, that the single cause is the cosmetic defect.

Another important point related with the validity of the WRVAS instrument is to analyze whether the cosmetic aspects the test refers to are actually those that cause the most concern. A look at other studies seems to verify this. Raso et al. [21] took photographs of the trunk of patients with idiopathic scoliosis and presented them to seven assessors who scored them according to the severity of various anomalies. Asymmetry of the scapulae, the shoulders and the waist explained 75% of the overall perception of deformity. Bridwell et al. [1] administered a questionnaire to patients pending surgery for idiopathic scoliosis, in which they were asked to score the aesthetic alterations that had motivated them to undergo surgery. Patients graded from greater to lesser severity their shoulder asymmetry, rib prominence, waist asymmetry, trunk imbalance, and thoracic asymmetry. Pratt et al. [22] asked patients scheduled for idiopathic scoliosis surgery to assess various aspects of the deformity. The following variables were cited as the most severe in their opinion: curvature of the column, rib hump, shoulder imbalance, waist asymmetry and trunk imbalance. The data provided by these studies seem to demonstrate that the aspects assessed by the WRVAS are, in general terms, adequate. To review: the aspects included in the scale are spinal deformity, rib prominence, flank prominence, thoracic deformity, trunk imbalance, and asymmetry of the shoulders and scapulae. With the exception of waist asymmetry, the remaining anomalies are the same as those used by other authors. It is likely that waist asymmetry would be assessed with the figures corresponding to trunk imbalance. In the bivariate analysis the highest correlation coefficients between the WRVAS and the SRS-22 self-image scale corresponded to the questions on spinal deformity (item 1, r = -0.55), trunk imbalance (item 5, r = -0.5) and scapular asymmetry (item 7, r = -0.48). The multivariate analysis confirmed that questions 1 and 5 have a significant influence in the SRS-22 self-image scale, explaining 39.5% of the variation. On the basis of these results, it seems that SRS-22 self-image recovers data that are not directly related to the trunk deformity, but rather to the social consequences of the perception of the deformity. From this viewpoint the two scales would not be mutually exclusive, but instead, complementary.

The scope of this study is limited to the internal consistency and construct validity of the instrument. A complete validation procedure would include an analysis of other crucial properties such as reliability and sensitivity to change in the patient's status. Another limitation is derived from the lack of stratification by curve patterns. The finding of different scores among the different curve patterns (thoracic/lumbar) would improve the validity of the WRVAS. Nevertheless, we feel that this preliminary assessment is a practical point of departure upon which to base further efforts. In our opinion, it could be interesting to assess the differences in perception of the deformity between patients and external evaluators. Sanders et al. [8] have already shown a good correlation between the scores given by patients and by their parents. The differences between patient and physician assessment remain to be investigated. Another worthwhile effort would be to determine what magnitude of the curve should correspond to the each grade of severity expressed in the figures. Evaluation of the degree of difference between the objective and perceived measure might be an important attribute of the scale, which would provide information on whether the deformity is exaggerated or attenuated in the patient's mind.

## Conclusion

Our results confirm the cosmetic compromise caused by the deformity and the significant subjective perception patients have of this compromise. The WRVAS seems to be a useful instrument for quantifying the perception patients have of their deformity. The metric properties analyzed (floor and ceiling effects, internal consistency and construct validity) were adequate and the instrument is valid. Future research in this line should continue with in-depth analysis of the relationship between the WRVAS questions and the radiological and clinical parameters of the deformity in order to confirm that each question actually measures the trait it is designed to measure.

## Competing interests

The author(s) declare that they have no competing interests.

## Authors' contributions

SP contributed in acquisition of data

JB contributed in analysis, interpretation of data and drafting the manuscript

CG contributed in revising the manuscript

JMC contributed in analysis, interpretation of data and drafting the manuscript.
